# An asymptomatic huge primary retroperitoneal pseudocyst: a case report and review of the literature

**DOI:** 10.1186/s12893-022-01510-5

**Published:** 2022-02-16

**Authors:** Lotfolah Abedini, Reza Hosseinpour, Saadat Mehrabi, Safoora Hejazinia, Mohammad Javad Yavari Barhaghtalab

**Affiliations:** 1 Department of General Surgery, Shahid Beheshti Hospital, Yasuj University of Medical Sciences, Yasuj, Iran; 2 Department of Pathology, Shahid Beheshti Hospital, Yasuj University of Medical Sciences, Yasuj, Iran

**Keywords:** Asymptomatic, Huge, Primary retroperitoneal pseudocyst, Review of the literature

## Abstract

**Background:**

Retroperitoneal non-pancreatic or idiopathic pseudocysts are very rare lesions. This case report aimed to present our patient and to check all the available literature on this kind of rare disease.

**Case presentation:**

Our patient was a 67-year-old Iranian man admitted with mild abdominal discomfort for three months. Ultrasonography and CT scan revealed a huge cystic structure within the retroperitoneal space. The lesion was excised through midline laparotomy and opening of the retro-peritoneum. The histopathology of the cyst wall revealed a benign cystic lesion with no epithelial lining. A histologic diagnosis of non-neoplastic retroperitoneal pseudocyst was made.

**Conclusion:**

The primary non-pancreatic retroperitoneal pseudocysts are rare lesions and have to be distinguished from other differential diagnoses of retroperitoneal lesions, and a surgeon should be aware of the possible occurrence of these lesions with unknown origin. Surgical excision is the only way to exclude malignancy and confirm the diagnosis.

## Background

The retro-peritoneum is a space situated behind the parietal peritoneum and in front of the transversalis fascia [1]. The retroperitoneum consists of three parts: the anterior pararenal space, the perirenal space, and the posterior pararenal space [1, 2]. The anterior pararenal space contains pancreas, 2nd to 4th parts of the duodenum, and the ascending and descending colon. The perirenal space contains the kidneys, proximal ureters, adrenal glands, and perirenal fat. The posterior pararenal space contains fat tissue and join inferiorly to the pelvic extraperitoneal space [2].

Most of the retroperitoneal masses originate from the retroperitoneal organs and are not considered as the primary retroperitoneal masses. A primary retroperitoneal mass is diagnosed once the location is inside the retroperitoneal space and after exclusion of the originity from an organ [2]. Primary retroperitoneal masses can be divided into solid and cystic groups and these two groups can be classified as neoplastic and non-neoplastic subgroups. Table 1 shows the differential diagnosis of the primary retroperitoneal masses [2, 3].

**Table 1:** Primary retroperitoneal masses (differential diagnosis)

Solid							Cystic	
Neoplastic						Non-neoplastic	Neoplastic	Non-neoplastic
Lymphoid tumors; lymphoma						Retroperitoneal fibrosis	Mature teratomas	Pancreatic pseudocysts
Sarcomas						Extra-medullary hematopoiesis	Mucinous cystadenomas	Non-pancreatic (Idiopathic) pseudocysts
Liposarcoma		Malignant fibrous histiocytoma		Leiomyosarcoma		Erdheim-Chester disease	Cystic mesotheliomas	Lymphoceles
Neurogenic tumors							Cystic lymphangioma	Urinomas
Schwannoma	Paraganglioma		Ganglioneuroma		Neurofibroma		Mullerian cysts	Hematomas
Immature teratomas							Epidermoid cysts	
							Tailgut cysts	
							Bronchogenic cyst	
							Pseudomyxoma retroperitonei	
							Perianal mucinous carcinoma	
							Cystic change in solid neoplasms	

Primary retroperitoneal cysts are structures not originating from any retroperitoneal organs and are very rare. They reach large sizes before causing any symptom and are often discovered accidentally [4–6]. The exact pathogenesis is unknown, but many possible pathologic mechanisms have been proposed and are divided into urogenital, mesocolic, teratomatous, parasitic, traumatic, and lymphatic types [5]. In the urogenital hypothesis, these tumors are originated from the remnants of the embryonal urogenital system, which include tissues of both epithelial and mesothelial origin [4, 6].

Though vague abdominal pain and distension are present in half of the cases, there are no clinical signs of retroperitoneal cysts in half of the patients, and they are diagnosed accidentally [6]. They may occasionally present with acute abdominal pain if they become hemorrhagic or infected [3–6]. Diagnosis is made with the use of ultrasonography and computed tomography (CT) scans. The walls of pseudocysts consist of dense fibrous tissues (the mesothelium) or mesonephric tissue with no epithelial lining [3–5].

Most retroperitoneal pseudocysts are originating from the pancrease [5]. Pancreatic pseudocyst contain pancreatic fluid, which is a complication of acute pancreatitis [2, 3]. Non-pancreatic pseudocysts have some charactersitics: a thick, fibrous wall or capsule, containing blood, pus, or serous fluid, and the cystic fluid is not associated with high levels of amylase or lipase [3, 6]. The characteristics of non-pancreatic pseudocysts are displayed on CT scans as unilocular or multilocular fluid-filled structures with the thick walls [5].

Non-pancreatic or idiopathic pseudocysts are rare lesions. This case report aimed to present our patient and to check all the available literature on this kind of rare disease.

## Case presentation

A 67-year-old Iranian man was admitted to Shahid Beheshti hospital affiliated with Yasuj University of Medical Sciences with mild lower abdominal discomfort (constant, dull abdominal aching) with a three-month duration. His bowel habits were normal, and he had no urgency, hesitancy, weak stream, or burning sensation when urinating. There was no fever, body weight loss, or recent history of trauma, surgery and chronic pancreatitis, or other diseases of the pancreas. Before presenting to the hospital, the patient visited other healthcare facilities and clinics and had received some unspecific oral medications like pantoprazole and hyoscine hydrobromide. His vitals were within normal range. A proper abdominal physical examination including inspection, auscultation, palpation, and percussion was done; the abdomen was soft, non-distended, without a palpable mass. The digital rectal exam was normal.

**Fig. 1:**
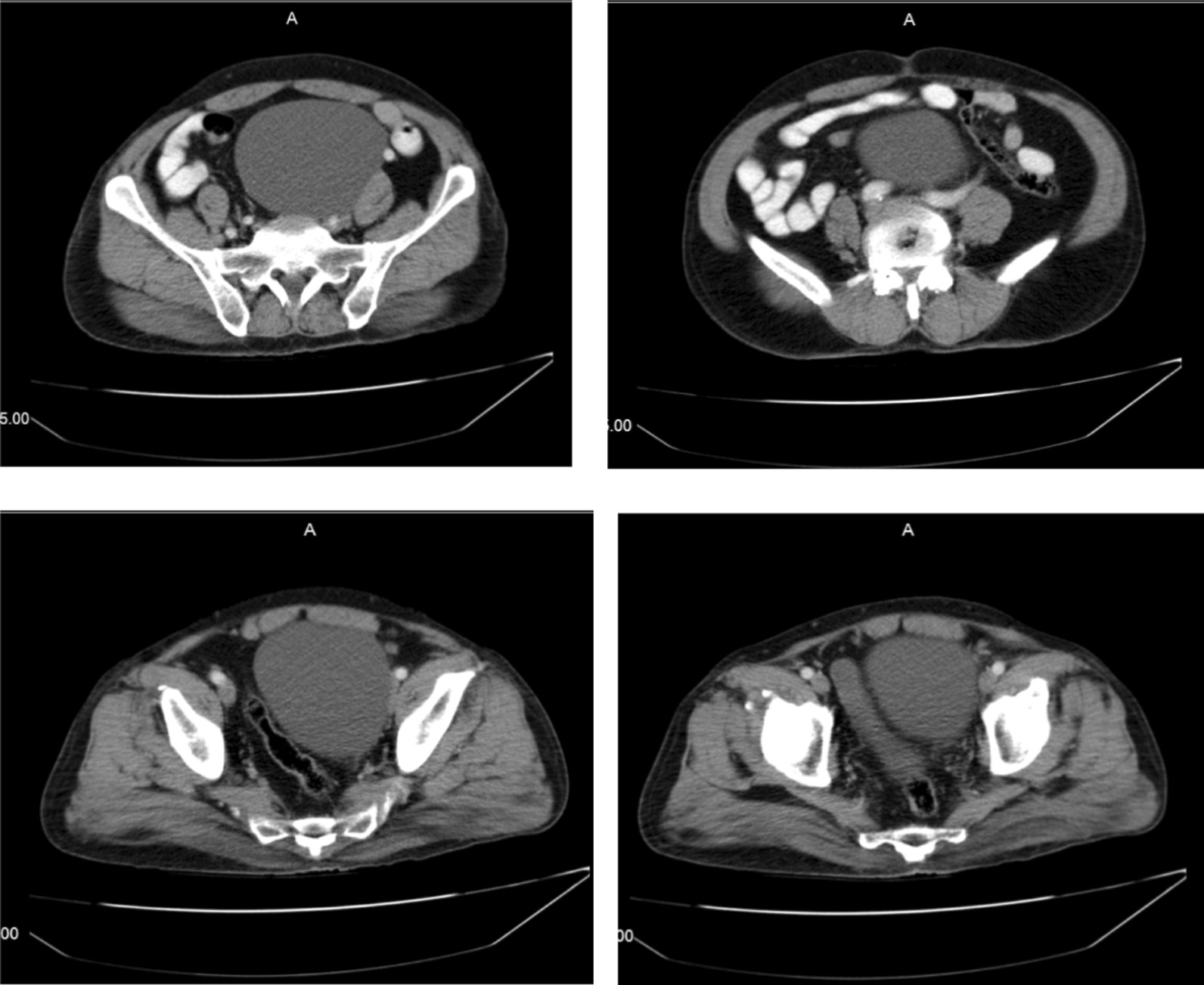
A contrast-enhanced CT scan of the abdomen and pelvic, a well-defined huge (140 × 120 mm) cystic structure within the left hemi-pelvic cavity

Ultrasonography of the abdomen showed a large retroperitoneal, cystic structure measuring 135 × 88 mm in mid-line position in the level of bifurcation of the aorta, and the urinary bladder was well distended with mildly increased wall thickening (5.5 mm) associated with some trabeculation in the wall without any stone. As the patient had no history of abdominal dysfunction, and there was no suspicion for bowel obstruction, foreign bodies, and urolithiasis, abdominal radiography was not ordered. However, contrast-enhanced CT scan of the abdomen and pelvic revealed a well-defined huge (140 × 120 mm), unilocular, thickened wall cystic structure within the left hemi-pelvic cavity extended from recto-sigmoid junction to the level of upper end of iliac crests and crossing mid-line, as well as with pressure effect over recto-sigmoid, lower rectus muscle, and urinary bladder. The cystic structure showed no solid components or gross internal septations and the content was homogenous. There was no overt connection the cystic structure and the surrounding organs (Fig. 1). The radiologist reported some differential diagnoses for this huge cystic structure as neuro-entric cysts, anterior sacral meningocele, and other cystic structures of the pelvic cavity. Paraclinical and laboratory findings as complete blood count (CBC), plasma concentrations of pancreatitis (amylase, lipase) and neoplasm markers (carcinoembryonic antigen (CEA), alpha fetoprotein (AFP), Cancer antigen (CA) 15–3, CA 19–9, and CA 125), urea, creatinine, bilirubin, aminotransferases, alkaline phosphatase and gamma-glutamyl transferase (GGT) were within normal range.

As this retroperitoneal cystic structure did not cause ureteric obstruction leading to obstructive uropathy, early preoperative ureteric stenting was not indicated according to the preoperational consult to the urologist.

**Fig. 2:**
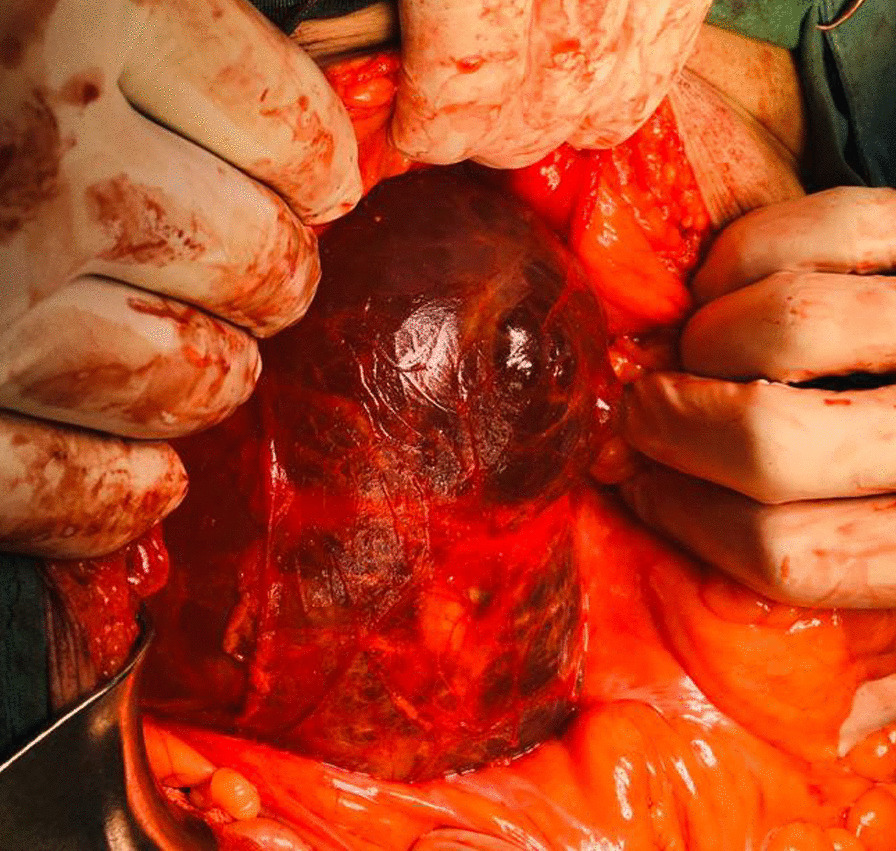
Intraoperative image of a large thick-walled retroperitoneal cyst

The patient underwent explorative laparotomy, and after opening into the retro-peritoneum, a large thick-walled retroperitoneal cyst, compressing the bladder wall was found (Fig. 2), afterward connections and adhesions of the mentioned structure to the adjacent organs such as recto-sigmoid colon, both ureters, iliac arteries, and the sacrum were released, and the structure was isolated and was removed successfThe mass was incised and contained clear serous fluid (pale yellow). The cytologic findings showed acellular cystic fluid with no malignant or epithelial cells. Cyst fluid culture was negative. One of our differential diagnoses was hydatid cyst which was ruled out by the absence of daughter cysts and normal echinococcal titers.

**Fig. 3:**
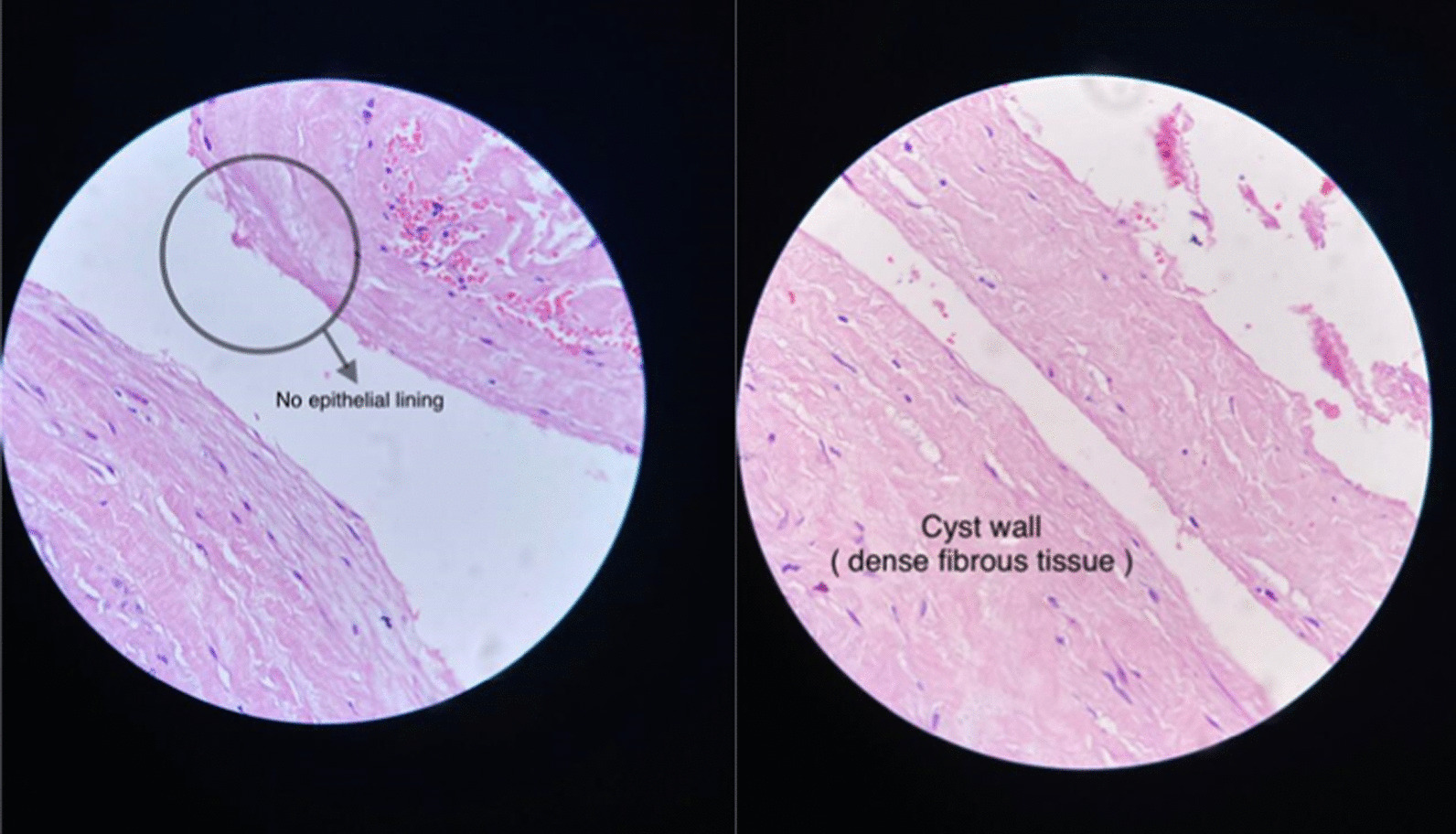
Histopathology of the cyst wall shows dense fibrous tissue, and no epithelial lining is present. A non-neoplastic retroperitoneal pseudocyst

The histopathology of the cyst wall revealed a benign cystic lesion with no epithelial lining. A histologic diagnosis of non-neoplastic retroperitoneal pseudocyst was made (Fig. 3).

The patient got per os (PO) on the 1st day after the operation, and became pain-free, had passed stool, had acceptable urine output on the second day after the operation, so then was discharged home on day 3 post-operation. The patient was followed up in the clinic with a normal abdominal examination and well-healing wounds after one week, and one month after discharge from the hospital, and there has not been any evidence of recurrence after 3 months of follow-up. He is optimistic about the future and says that this event has helped him to pay more attention to his health afterward.

## Discussion

A retroperitoneal non-pancreatic pseudocyst is a rare surgical entity that carries a range of differential diagnoses with the incidence rate of 1 in 5750 to 1 in 250,000 [3]. In this regard, all previously published reports on the cases with idiopathic or primary retroperitoneal pseudocyst were searched and found on the web and only available full-text original articles were met. As a result, thirteen previous cases were elucidated ad after adding our case report to them, a total of fourteen cases were evaluated and summarized in Table 2 according to the originated country, the published year of the case report, patient’s gender, age, chief complaints, physical examinations, ultrasound, CT scan, magnetic resonance imaging (MRI), and other radiological findings (if done), surgery, and pathologic results [3–16].

**Table 2:** Published reports on the cases with idiopathic or primary retroperitoneal pseudocyst

	Year and Country	Sex/ Age	Chief Complaints	Physical Examination	Ultrasound, magnetic resonance (MR) imaging, and other radiological findings	CT scan findings	Surgery	Pathology
1	Japan, 1978 [7]	F/54	The patient was hospitalized for the management of diabetes mellitus	A solid mass was felt in the left upper quadrant at an abdominal examination	An abdominal scout film and excretory urography showed a grape-fruit sized mass unrelated to the urinary tract	Not mentioned	The tumor removed was 7 × 6 × 8.5 cm in size, and 240 g in weight	The content of the tumor was gray necrotic material. The histology revealed a fibrous capsule and degenerative material containing cholesterin crystals
2	India, 1995 [8]	F/3	A mass in the left side of the abdomen noticed by the parents 10 days prior to the admission	A well-defined, non-tender, irregular mass in the left lumbar region with restricted mobility	Ultrasound: Multi-cystic retro-peritoneal mass anterior to left kidney that displaced the left kidney upward	Not mentioned	Exploration, completely excision of the cyst	The absence of an epithelial lining, the wall was composed of collagen and fibrin with inflammatory-cell infiltration, a retroperitoneal pseudocyst
3	Spain, 1996 [9]	M/48	Dysuria and urinary frequency	On digital rectal examination, a right-side mass was detected	1. Endo-rectal ultrasound showed a 13-cm well-defined retro-rectal mass with posterior acoustic enhancement, 2. A barium enema showed the compression of the rectum, without mucosal changes or communication between the cyst and the rectum lumen	A pre-sacral cystic mass with thin walls and peripheric calcifications. The rectum was displaced anteriorly	A cystic pelvic mass surrounded by fat was resected	Brownish fibrous tissue without epithelium in the wall
4	India, 2007 [4]	F/80	Vague ‘dragging’ pain in the left upper quadrant	A large mass in the left hypochondrium, mobile and non-tender	Ultrasound: A large retroperitoneal, cystic mass, 19 × 17 cm with a calcified wall	CT scan of the abdomen confirmed the ultrasound findings	Laparoscopic excision, four ports	No lining epithelium, extensive calcification and chronic inflammatory cells, primary retroperitoneal pseudocysts
5	UK, 2008 [10]	M/51	Right-sided abdominal pain for three days, jaundice, fever	jaundice, fever	Fluid collection/mass measuring 14 cm in the right hepato-renal space, separated from the liver, pancreas and the right kidney, lack of definition of the right suprarenal gland	As the same finding as sonography	1.150 ml brown-colored fluid was aspirated from the mass under CT guidance, 2. laparotomy for excision of the mass and right adrenalectomy	Normal adrenal gland was adherent by fibrous tissues to the external wall of the cyst, but the cyst was not arising from the adrenal. The cyst wall consisted of a thick layer of fibrous tissues which showed focal calcifications and areas of acute and chronic inflammation, no epithelial lining, granulation tissue as a part of the lining of the cyst, idiopathic benign retroperitoneal cyst
6	USA, 2009 [11]	M/59	Sudden onset of left upper quadrant abdominal pain, nausea, and vomiting	Involuntary guarding, rebound, and tenderness over the epigastrium and left upper quadrant	1. Ultrasound was not mentioned, 2. MR imaging showed dynamic fat-suppressed T1-weighted image following intravenous gadolinium injection and enhancement of the wall of the cystic mass	A 5.0 × 3.8 cm, well marginated, rounded mass in the left upper quadrant, within the small bowel mesentery	Elective exploratory laparotomy	The cyst wall was lined by fibrous tissue, with no evidence of an endothelial lining, and showed chronic inflammation with lipid clefts and calcification, which are indicative of prior rupture (a non-pancreatic pseudocyst of the mesentery that had undergone focal rupture)
7	Poland, 2011 [6]	F/27	Abdominal discomfort	A vague right side abdominal, non-tender mass	Not mentioned	A huge, unilocular, right-sided retroperitoneal cystic mass, unknown origin, well-demarcated margins, extending from the liver to the pelvis, dislocations of the right kidney and adrenal gland	Laparotomy, exploration, and totally excision of the cyst	Absence of an epithelial lining, dense connective tissue with focal inflammatory cell infiltration primary retroperitoneal pseudocyst
8	Taiwan, 2012 [5]	M/43	Progressive lower abdominal pain, abdominal distension and frequent urination	A large mass in the lower abdomen, soft, fixed, and non-tender	Not mentioned	A large thick-walled retroperitoneal cyst compressing the bladder	Laparotomy, vertical midline incision, peritoneal approach	Dense fibrous tissue with no epithelial lining, non-pancreatic pseudocyst
9	India, 2013 [12]	M/76	Abdominal pain over the right upper quadrant and constipation	A mass effect protruding out of the right mid-abdomen, measuring 15 × 15 cm, non-tender, non-mobile and not moving with respiration	Not mentioned	A large well defined thin-walled cystic lesion measuring 10.3 × 13.9 × 14.3 cm in the right lumbar and iliac region without calcification or hemorrhage	The cyst was excised in toto after separating it from the duodenum, transverse colon, and the ureter	Absence of epithelia and was reported as pseudocyst
10	China, 2016 [13]	F/27	Presented with the discovery of a cyst in the left upper quadrant of six years duration and abdominal distention of ten days	On visual inspection of the abdomen, there was a mass effect protruding out of the left upper abdomen. Abdominal physical examination revealed a large mass was non-mobile and non-tender	1. Ultrasound: A large retroperitoneal, cystic mass measuring 11 cm × 14 cm with a thick wall, 2. MRI of the abdomen confirmed the ultrasound findings	A contrast-enhanced CT scan of the abdomen confirmed the ultrasound findings	Four ports laparoscopy, with puncture and aspiration of the cyst, then a complete excision of the cyst using a combination of blunt and sharp dissection	The cyst wall was devoid of lining epithelium with extensive inflammatory cells and multinucleate giant cells, confirming the diagnosis of a pseudocyst
11	The UK, 2016 [14]	M/70	Bilateral reducible groin swellings	Incidental finding of a large right-sided cystic mass below the liver edge. This was noted clinically when the patient was on the table and was confirmed laparoscopically	Not mentioned	A unilocular, cystic lesion, 22 × 20 × 19 cm arising from within the right side of the abdomen	Laparotomy and excision of the cyst using both blunt and sharp dissection	The cyst wall was lined by foamy macrophages and fibrin, with some chronic inflammation in the wall. An epithelial or mesothelial lining was absent, a benign non-pancreatic pseudocyst
12	India, 2019 [15]	M/53	Abdominal distension, pain, reduced appetite, and intermittent fever	Tachycardia, mild tachypnea, and had tenderness in the epigastric region with a palpable lump in the epigastrium, right hypochondrium and extended into the right iliac fossa	Ultrasound: A large cystic lesion in the epigastrium	A large cystic lesion in close vicinity of the pancreatic head and neck, extending into sub-hepatic space, pushing the transverse colon down and reaching up to the right iliac fossa	1. EUS guided cystogastrostomy with the placement of SEMS, 2. Laparotomy due to failure of symptomatic resolution after endoscopic management, a right subcostal incision (A Cattell-Braasch maneuver)	Cyst lined by a fibrino-purulent exudate, and no epithelial lining, wall contained proliferating granulation tissue and fibroblasts, chronic inflammation
13	Australia, 2019 [16]	M/55	An incidental finding on CT of a’large adrenal mass’	Not mentioned	Not mentioned	An incidental 40 × 32 mm mass positioned adjacent to the medial border of the spleen, and the left adrenal gland	An elective laparoscopy	A non- pancreatic fibrous pseudocyst, a thick calcified wall, the absence of epithelial lining, and widespread inflammatory change
14	Qatar, 2020 (3)	M/49	Right iliac fossa pain, constipation	The abdomen was non-tender, non-distended, and soft to touch, no definite mass palpated	Not mentioned	A 7 × 6 cm cystic lesion, incomplete peripheral calcification in the pelvis	Laparoscopic cyst excision	No epithelial or endothelial lining, idiopathic retroperitoneal non-pancreatic pseudocyst
15	Iran, 2020 (current study)	M/67	Mild abdominal discomfort for 3 months	The abdomen was non-tender, non-distended, and soft to touch, no definite mass palpated	Ultrasound: A large retroperitoneal, cystic structure measuring 135 × 88 mm in mid-line position in the level of bifurcation of the aorta	A well-defined huge (140 × 120 mm), unilocular, thickened wall cystic structure within the left hemi-pelvic cavity with pressure effect over recto-sigmoid, lower rectus muscle, and urinary bladder	Laparotomy, total excision	Benign cystic lesion with no epithelial lining, a primary non-neoplastic retroperitoneal pseudocyst

Of the total 15 reports, 4 reports were for India, and that was amazing. Males were the dominant sex encountered in 10 cases. The mean age was 50.7857 ± 10.977 with a 95% confidence interval. The youngest patient was a 3-year-old girl and the oldest was an 80-year-old woman. Physical examination was not mentioned in one, and in two patients, the abdomen was non-tender, non-distended, and soft to touch, and no definite mass palpated, but in the majority of the cases (10 patients), a palpable mass was detected 9 cases in the abdominal, and one in the rectal examination. Abdominal radiography was used as the only diagnostic modality in one case and showed a grape-fruit-sized mass. Trans-abdominal ultrasound was used in 6 cases and revealed a retroperitoneal cystic/mass lesion in 5 patients and a multi-cystic lesion in one patient (ultrasound was the only diagnostic modality used in this case). Endo-rectal ultrasound was used in one patient and showed a retro-rectal mass. CT scan was the most common modality used in detecting the pathology and was used in 13 patients. MRI was used in two patients and confirmed the ultrasound and CT scan findings in one patient each retrospectively. Barium enema was done in only one case and showed the compression of the rectum by the lesion in this case.

Location of the cystic structure was reported to be in the pelvic cavity in 2 patients, retro-peritoneum in 3, and left upper quadrant of the abdomen within the small bowel mesentery, just mentioned right-sided, right-sided with the extension from the liver to the pelvis, right-sided lumbar and iliac region, pancreatic head and neck with the extension into the sub-hepatic space, in the right hepato-renal space, adjacent to the medial border of the spleen, and the left adrenal gland in one patient each retrospectively. Dislocation or displacement of the viscera was seen in the right kidney and adrenal gland, rectum, and transverse colon in one patient each retrospectively. Pressure effect of the lesion was seen over the urinary bladder in two cases, and recto-sigmoid, and lower rectus muscle in one patient each retrospectively.

Laparoscopic surgery was performed in four cases [3, 4, 12, 15], the other majority of the cases were operated through an open approach with total resection of the lesion [5–14, 16]. Endoscopic ultrasonographic (EUS) guided cystogastrostomy with the placement of a self-expandable metallic stent (SEMS) was done in one patient [14]. The absence of an epithelial lining was seen in the majority of the patients (13 cases) [3–6, 8–16].

The retroperitoneal pseudocysts are asymptomatic before reaching a large size, and compress over the adjacent structures, and are often diagnosed accidentally [6, 12]. There seem to be two theories for the occurrence of such an enlarged cyst in the retroperitoneal cavity: 1. the retroperitoneal space contains organs originating from the ectoderm and endoderm and are surrounded by a loose network of connective tissue. In this setting, both primary and metastatic tumors grow silently and become symptomatic when they become so large. If they originate from the Wolffian duct, we would see clear fluid, and if they are teratomatous, sebaceous material would be seen [4], 2. Because there is no mesothelial lining within the retroperitoneal space, the extra fluid cannot be reabsorbed and the pseudocysts usually make much volume [6].

Complete excision is a cure for these retroperitoneal cysts. Marsupialization and partial excision are not recommended, because recurrence is common. The surgical methods are conventional laparotomy (intraperitoneal approach), extraperitoneal approach, and transperitoneal flank approach [5], although the laparoscopic approach has been reported either [5–12, 14, 16]. We used the conventional method because the size of the cyst was very large, and the cyst has to be dissected from the other retroperitoneal structures and adhesions without being ruptured.

## Conclusion

Primary non-pancreatic retroperitoneal pseudocysts are very rare lesions and have to be distinguished from other differential diagnoses of retroperitoneal lesions, and a surgeon should be aware of the possible occurrence of these lesions with unknown origin. Surgical excision is the only way to exclude malignancy and confirm the diagnosis.

## Data Availability

The datasets used and/or analysed during the current study are available from the corresponding author on reasonable request.

## Abbreviations

AFP: Alpha fetoprotein
CA: Cancer antigen
CEA: Carcinoembryonic antigen
CT: Computed tomography
CBC: Complete blood count
EUS: Endoscopic ultrasonographic
GGT: Gamma-glutamyl transferase
MRI: Magnetic resonance imaging
PO: Per os
SEMS: Self-expandable metallic stent
